# Supplementary material for: A Toll-receptor map underlies structural brain plasticity
Source: eLife. 2020 Feb 18;9:e52743. doi: 10.7554/eLife.52743 (PMC7077983; doi:10.7554/eLife.52743)
Supplement: Supplementary file 1. [file elife-52743-supp1.docx]

| **Supplementary File 1. Key Resources Table** | | | | |
| --- | --- | --- | --- | --- |
| Reagent type (species) or resource | Designation | Source or reference | Identifiers | Additional information |
| gene (Drosophila melanogaster) | Toll-1 | Flybase | FBgn0262473 |  |
| gene (Drosophila melanogaster) | Toll-2 | Flybase | FBgn0004364 |  |
| gene (Drosophila melanogaster) | Toll-3 | Flybase | FBgn0015770 |  |
| gene (Drosophila melanogaster) | Toll-4 | Flybase | FBgn0032095 |  |
| gene (Drosophila melanogaster) | Toll-5 | Flybase | FBgn0026760 |  |
| gene (Drosophila melanogaster) | Toll-6 | Flybase | FBgn0036494 |  |
| gene (Drosophila melanogaster) | Toll-7 | Flybase | FBgn0034476 |  |
| gene (Drosophila melanogaster) | Toll-8 | Flybase | FBgn0029114 |  |
| gene (Drosophila melanogaster) | Toll-9 | Flybase | FBgn0036978 |  |
| gene (Drosophila melanogaster) | MyD88 | Flybase | FBgn0033402 |  |
| gene (Drosophila melanogaster) | Sarm | Flybase | FBgn0262579 |  |
| gene (Drosophila melanogaster) | Dorsal | Flybase | FBgn0260632 |  |
| gene (Drosophila melanogaster) | Dif | Flybase | FBgn0011274 |  |
| gene (Drosophila melanogaster) | wek | Flybase | FBgn0001990 |  |
| gene (Drosophila melanogaster) | TrpA1 | Flybase | FBgn0035934 |  |
| gene (Drosophila melanogaster) | Rbf | Flybase | FBgn0015799 |  |
| gene (Drosophila melanogaster) | Pi3K92E | Flybase | FBgn0015279 |  |
| gene (Drosophila melanogaster) | JNK | Flybase | FBgn0000229 |  |
| gene (Drosophila melanogaster) | Cactus | Flybase | FBgn0000250 |  |
| gene (Drosophila melanogaster) | Yorkie | Flybase | FBgn0034970 |  |
| gene  (Baculovirus) | p35 |  | FBgn0014459 |  |
| Strain (Escherichia coli) | DH5α | New England BioLabs | C2987I | Chemically competent |
| Strain (Escherichia coli) | 10-beta | New England BioLabs | C3019I | Chemically competent |
| genetic reagent (D. melanogaster) | Toll-2^pTV^ | This paper |  | See “Molecular Biology” in Materials and Methods Baena-Lopez, Alexandre et al. 2013 Development |
| genetic reagent (D. melanogaster) | Df(2R)BSC594 | Bloomington Drosophila Stock Center (BDSC) | BDSC:25678;  FLYB:FBst0025678  RRID:BDSC_25678 |  |
| genetic reagent (D. melanogaster) | Df(2R)BSC22 | BDSC | BDSC:6647; FLYB:FBst0006647 RRID:BDSC_6647 |  |
| genetic reagent (D. melanogaster) | 18w^D7-35^ | BDSC | BDSC:4372  FLYB: FBst0004372  RRID:BDSC_4372 |  |
| genetic reagent (D. melanogaster) | Toll-3 GAL4^MI02994^ | This paper | Toll-3^MI02994^ RRID:BDSC_35889 | Based on method described in  Venken et al. 2011 |
| genetic reagent (D. melanogaster) | Toll-6 GAL4^MIO2127^ | This paper | Toll-6^MI02127^ RRID:BDSC_34467 | As above |
| genetic reagent (D. melanogaster) | Toll-7 GAL4^MI13963^ | This paper | Toll-7^MI13963^ RRID:BDSC_59468 | As above |
| genetic reagent (D. melanogaster) | Toll-8GAL4^MD806^ | BDSC | BDSC:36548 FLYB:FBst0036548  RRID:BDSC_36548 |  |
| genetic reagent (D. melanogaster) | MyD88GAL4^NP6394^ | Kyoto Stock Centre (DGRC) | DGRC: 105229 FLYB: FBst0304088 |  |
| genetic reagent (D. melanogaster) | sarm-GAL4^NP7460^ | DGRC | DGRC:105471 FLYB: FBst0304329 |  |
| genetic reagent (D. melanogaster) | Sarm-GAL4^NP0257^ | DGRC | DGRC:103571 FLYB: FBst0302440 |  |
| genetic reagent (D. melanogaster) | Dif-GFP.FPTB | BDSC | BDSC:42673 FLYB: FBst0042673  RRID:BDSC_42673 |  |
| genetic reagent (D. melanogaster) | Dl-GFP-FPTB | BDSC | BDSC:42677 FLYB:FBst0042677 RRID:BDSC_42677 |  |
| genetic reagent (D. melanogaster) | wek GFP-FLAG-FPTB^VK00033^ | BDSC | BDSC: 67719 FLYB:FBst0067719  RRID:BDSC_67719 |  |
| genetic reagent (D. melanogaster) | TRE-DsRED | BDCS | BDCS59011  FLYB:FBti0147636  RRID:BDSC_59011 | Gift from Yun Fan; JNK signalling reporter |
| genetic reagent (D. melanogaster) | TRE-DsRED | BDCS | 59012  FLYB:FBti0147635  RRID:BDSC_59012 | Gift from Yun Fan; JNK signalling reporter |
| genetic reagent (D. melanogaster) | UAS Flybow 1.1 | BDSC | BDSC: 35537 FLYB: FBst0035537  RRID:BDSC_35537 | Gift from  I. Salecker |
| genetic reagent (D. melanogaster) | 10xUAS-myr-td-Tomato | Gift from B. Pfeiffer |  |  |
| genetic reagent (D. melanogaster) | UAS-histone-YFP | Hidalgo Lab |  | Forero, Kato & Hidalgo (2012) J Microscopy 246, 202-12 |
| genetic reagent (D. melanogaster) | UAS Toll-2^EP-709^ | BDSC | BDSC: 43442  FBti0101281  RRID:BDSC_43442 |  |
| genetic reagent (D. melanogaster) | UAS-TrpA1 | BDSC | BDSC:26263 FLYB: FBst0026263  RRID:BDSC_26263 |  |
| genetic reagent (D. melanogaster) | UAS Toll-2^attP2^ | This paper | Toll-2 CDS: FBpp0085620 |  |
| genetic reagent (D. melanogaster) | UAS Wek | FlyORF | FlyORF:F000070 FLYB:FBst0501584 |  |
| genetic reagent (D. melanogaster) | UAS Rbf^280^ | BDSC | BDSC:50748 FLYB:FBti0152614  RRID:BDSC_50748 |  |
| genetic reagent (D. melanogaster) | UAS p35 | Hidalgo Lab | FBgn0014459 |  |
| genetic reagent (D. melanogaster) | UAS-Dp110CAAX | BDSC | BDSC:25908 FBst0025908  RRID:BDSC_25908 |  |
| genetic reagent (D. melanogaster) | UAS-Toll-1RNAi | BDSC | BDSC:31044 FBti0130909  RRID:BDSC_31044 |  |
| genetic reagent (D. melanogaster) | UAS-Toll-1RNAi | BDSC | BDSC:31477 FBti0130724  RRID:BDSC_31477 |  |
| genetic reagent (D. melanogaster) | UAS-Toll-2RNAi | BDSC | BDSC:30498 FBti0128524  RRID:BDSC_30498 |  |
| genetic reagent (D. melanogaster) | UAS-Toll-2RNAi | VDRC | VDRC:36305 FBst0471685 |  |
| genetic reagent (D. melanogaster) | UAS-Toll-2RNAi | VDRC | VDRC: 44386  FBst0465553 |  |
| genetic reagent (D. melanogaster) | UAS-Toll-6RNAi | BDSC | BDSC: 56048 FBst0056048  RRID:BDSC_56048 |  |
| genetic reagent (D. melanogaster) | UAS-Toll-6RNAi | VDRC | VDRC:928 FBst0471444 |  |
| genetic reagent (D. melanogaster) | UAS-Toll-7RNAi | Fly Stocks of National Institute of Genetics (NIG-FLY) | NIG-FLY:8595R-2 |  |
| genetic reagent (D. melanogaster) | UAS-Toll-7RNAi | NIG-FLY | NIG-FLY:8595R-3 |  |
| genetic reagent (D. melanogaster) | UAS-Toll-8RNAi | BDSC | BDSC: 28519 FBst0028519  RRID:BDSC_28519 |  |
| genetic reagent (D. melanogaster) | UAS-Toll-8RNAi | VDRC | VDRC: 27098 |  |
| genetic reagent (D. melanogaster) | UAS-MyD88RNAi | VDRC | VDRC:25399 FBst0455867 |  |
| genetic reagent (D. melanogaster) | UAS-MyD88RNAi | VDRC | VDRC:25402 FBst0455868 |  |
| genetic reagent (D. melanogaster) | UAS-JNK-RNAi | VDRC | VDRC:34138 FBst0460476 |  |
| genetic reagent (D. melanogaster) | UAS-cactusRNAi | BDSC | BDSC: 34775 FBst0034775  RRID:BDSC_34775 |  |
| genetic reagent (D. melanogaster) | UAS-yorkie-RNAi | BDSC | BDSC:31965 FBst0031965  RRID:BDSC_31965 |  |
| genetic reagent (D. melanogaster) | UAS Wek RNAi | BDSC | BDSC: 35680 FBst0035680  RRID:BDSC_35680 |  |
| genetic reagent (D. melanogaster) | Yki-GFP | Gift from Nic Tapon & Barry Thompson |  | Fletcher, Diaz-de-la-Loza et al. 2018 Development |
| genetic reagent (D. melanogaster) | PCNA-GFP | Hidalgo Lab |  | Kato et al (2011) PLoS Biology 9, e1001133 |
| antibody | Anti-GFP (Rabbit polyclonal) | Molecular Probes | Cat# A-11122  RRID:AB_221569 | IF (1:250) |
| antibody | Anti-DsRed (Rabbit monoclonal) | Clontek | Takara Bio Cat# 632496, RRID:AB_10013483 | IF (1:100) |
| antibody | anti-Toll-1 (Goat) | Santa Cruz Biotechnology |  | IF (1:10) |
| antibody | Anti-FoXO (Rabbit) | Gift from  P. Léopold |  | IF (1:500) |
| antibody | Anti-Dpn (Guinea pig) | Gift from  J. Jan |  | IF (1:1000) |
| antibody | Anti-Miranda (Rat monoclonal) | Abcam | Cat# ab197788 | IF (1:10) |
| antibody | Anti-Repo (Mouse monoclonal) | Developmental Studies Hybridoma Bank (DSHB) | Cat# 8D12 RRID:AB_528448 | IF (1:10) |
| antibody | Anti-Elav (Rat monoclonal) | DSHB | Cat# 7E8A10 RRID: AB_528218 | IF (1:250) |
| antibody | Anti-Rabbit 488 (Donkey polyclonal) | Alexa Fluor | Molecular Probes Cat# A-21206, RRID:AB_141708 | IF (1:250) |
| antibody | Anti-Rabbit 546 (Goat polyclonal) | Alexa Fluor | Molecular Probes Cat# A-11030, RRID:AB_144695 | IF (1:250) |
| antibody | Anti-Guinea Pig 633 (Goat polyclonal) | Alexa Fluor | Innovative Research Cat# A21105, RRID:AB_1500611 | IF (1:250) |
| antibody | Anti-Rat 647 (Goat polyclonal) | Alexa Fluor | Molecular Probes Cat# A-21247, RRID:AB_141778 | IF (1:250) |
| antibody | Anti-Mouse 647 (Goat polyclonal) | Alexa Fluor | Molecular Probes Cat# A-21236, RRID:AB_141725 | IF (1:250) |
| recombinant DNA reagent | Toll-2^pTV^ (plasmid) | This paper |  | See “Molecular Biology” in Materials and Methods |
| recombinant DNA reagent | pTV^mCherry^ (plasmid) | Gift from J.P. Vincent |  | Baena-Lopez et al. 2013 Development |
| recombinant DNA reagent | pU6.3 Toll-2 gRNA (plasmid) | This paper |  | See “Molecular Biology” in Materials and Methods |
| recombinant DNA reagent | pCFD3-dU6:3gRNA (plasmid) | AddGene | Addgene Cat# 49410  RRID:Addgene_49410 | Port et al. 2014 |
| recombinant DNA reagent | pU6.3 Toll-4 gRNA (plasmid) | This paper |  | See “Molecular Biology” in Materials and Methods |
| recombinant DNA reagent | pU6.3 Toll-5 gRNA (plasmid) | This paper |  | See “Molecular Biology” in Materials and Methods |
| recombinant DNA reagent | attB2-Gal4 (plasmid) | Addgene | Addgene Cat#62896  RRID:Addgene_62896 | Diao et al. 2015 |
| recombinant DNA reagent | T2A-Gal4 (plasmid) | Addgene | Addgene  Cat# 125211  RRID:Addgene_125211 |  |
| recombinant DNA reagent | Toll-4 Gal4 (plasmid) | This paper |  | See “Molecular Biology” in Materials and Methods |
| recombinant DNA reagent | Toll-5 Gal4 (plasmid) | This paper |  | See “Molecular Biology” in Materials and Methods |
| recombinant DNA reagent | pUAS-gw-Toll-2 CDS (plasmid) | This paper |  | See “Molecular Biology” in Materials and Methods |
| recombinant DNA reagent | pUAS-gw-attB | Gift from  K. Basler |  |  |
| sequence-based reagent | Toll-2 3'HRF2 | This paper | PCR primers | ggactagtcc aaactccccctatggccata |
| sequence-based reagent | Toll-2 3'HRR N2 | This paper | PCR primers | ttggcgcgccaa gtgagggttgtacatagccac |
| sequence-based reagent | Toll-2 5'HRF2 | This paper | PCR primers | ataagaatgcggccgcggcaattagttaggcctgaa |
| sequence-based reagent | Toll-2 5'HRR2 | This paper | PCR primers | cggggtaccccgtttggtgatttgctagttgg |
| sequence-based reagent | Toll-2 gRNA SOM | This paper | PCR primers | gtcgctcctgcatgccagtgaac |
| sequence-based reagent | Toll-2 CDS attB Fwd | This paper | PCR primers | ggggacaagtttgtacaaaaaagcaggctATGCCAGCCACATCTTCCAT |
| sequence-based reagent | Toll-2 CDS attB Rev | This paper | PCR primers | ggggaccactttgtacaagaaagctgggtGACCAGGAAAGCTTGGCCGT |
| sequence-based reagent | Toll-4 gRNA S | This paper | PCR primers | gtcgATCCGTGCTGATAACTGAGA |
| sequence-based reagent | Toll-5 S BbsI gRNA | This paper | PCR primers | gtcGTACAACGATGCGCCCGGTT |
| commercial assay or kit | Gateway BP Clonase II Enzyme Mix | Invitrogen | Cat#: 11789020 |  |
| commercial assay or kit | Gateway LR Clonase II Enzyme Mix | Invitrogen | Cat#: 11791020 |  |
| commercial assay or kit | QIAquick Gel Extraction Kit | Qiagen | Cat No./ID: 28704 |  |
| commercial assay or kit | QIAGEN Plasmid Midi Kit | Qiagen | Cat No./ID: 12143 |  |
| software, algorithm | GraphPad Prism 6 | GraphPad |  |  |
| software, algorithm | DNASTAR Lasergene | DNASTAR |  |  |
| software, algorithm | DeadEasy-Central Brain | This paper |  | ImageJ Plugin  See “Microscopy, imaging and development of Deadeasy plug-ins” in Materials and Methods |
| software, algorithm | DeadEasy-  Optic Lobes | This paper |  | ImageJ Plugin  See “Microscopy, imaging and development of Deadeasy plug-ins” in Materials and Methods |
| software, algorithm | DeadEasy-Kenyon Cells | This paper |  | ImageJ Plugin  See “Microscopy, imaging and development of Deadeasy plug-ins” in Materials and Methods |
| software, algorithm | Adobe Illustrator | Adobe |  |  |
| software, algorithm | Adobe Photoshop | Adobe |  |  |
| software, algorithm | ImageJ, Fiji | NIH |  |  |
