# Supplementary material for: A Toll-receptor map underlies structural brain plasticity
Source: eLife. 2020 Feb 18;9:e52743. doi: 10.7554/eLife.52743 (PMC7077983; doi:10.7554/eLife.52743)
Supplement: Supplementary file 3. — Primers used for all cloning and RT-PCR experiments. [file elife-52743-supp3.docx]

**Supplementary File 3 List of primers used**

| Primer No. | NAME | GENE | PRIMER SEQUENCE(5'-->3') | DESCRIPTION |
| --- | --- | --- | --- | --- |
|  |  |  |  |  |
| 1 | Toll-2 3'HRF2 | Toll-2 | ggactagtcc aaactccccctatggccata | Forward primer to amplify Toll-2 3'HR, with SpeI cut site, cloning into PTV |
| 2 | Toll-2 3'HRR N2 | Toll-2 | ttggcgcgccaa gtgagggttgtacatagccac | Reverse primer to amplify Toll-2 3'HR, with AscI cut site, cloning into PTV |
| 3 | Toll-2 5'HRF2 | Toll-2 | ataagaatgcggccgcggcaattagttaggcctgaa | Forward primer to amplify Toll-2 5'HR, with NotI cut site, cloning into PTV |
| 4 | Toll-2 5'HRR2 | Toll-2 | cggggtaccccgtttggtgatttgctagttgg | Reverse primer to amplify Toll-2 5'HR, with KpnI cut site, cloning into PTV |
| 5 | Toll-2 gRNA SOM | Toll-2 | gtcgctcctgcatgccagtgaac | Toll-2 gRNA sense oligonucleotides for U6.3 |
| 6 | Toll-2 gRNA ASO | Toll-2 | aaacgttcactggcatgcaggagc | Toll-2 gRNA anti-sense oligonucleotides for U6 |
| 7 | Toll-2 CDS attB Fwd | Toll-2 | ggggacaagtttgtacaaaaaagcaggctATGCCAGCCACATCTTCCAT | Forward primers for amplification of Toll-2 CDS for gateway cloning, pUAST |
| 8 | Toll-2 CDS attB Rev | Toll-2 | ggggaccactttgtacaagaaagctgggtGACCAGGAAAGCTTGGCCGT | Reverse primers for amplification of Toll-2 CDS for gateway cloning, pUAST |
| 9 | Toll-4 5' HR R NotI | Toll-4 | ATAGTTTAGCGGCCGCATTCTTATGGTATTGTACCGCAGGGCTT | Reverse primer to amplify Toll-4 5'HR, with NotI cutting site, cloning into pGEM-T2A-GAL4 |
| 10 | Toll-4 5' HR F AgeI | Toll-4 | GTATCACCGGTGTGTGTGTTTGGGCTACGTG | Forward primer to amplify Toll-4 5'HR, with AgeI cutting site, cloning into pGEM-T2A-GAL4 |
| 11 | Toll-4 3' HR R SpeI | Toll-4 | GGACTAGTCCTATCGGGGAGTTCAGCCAAC | Reverse primer to amplify Toll-4 3'HR, with SpeI cutting site, cloning into pGEM-T2A-GAL4 |
| 12 | Toll-4 3' HR F AscI | Toll-4 | AGGCGCGCCTTAAGCCTACTTGGCATTCGA | Forward primer to amplify Toll-4 3'HR, with AscI cutting site, cloning into pGEM-T2A-GAL4 |
| 13 | Toll-4 gRNA S | Toll-4 | gtcgATCCGTGCTGATAACTGAGA | Sense gRNA oligo, cloning into pU6.3 |
| 14 | Toll-4 gRNA AS | Toll-4 | aaacTCTCAGTTATCAGCACGGAT | Antisense gRNA oligo to anneal to above, cloning into pU6.3 |
| 15 | Toll-5 5' HR F AgeI | Toll-5 | GTATCACCGGTtcctgttaaatcccctcgacc | Forward primer to amplify Toll-5 5'HR, with AgeI cutting site |
| 161 | Toll-5 5' HR R NotI | Toll-5 | ATAGTTTAGCGGCCGCATTCTTATTCTTCACTTCACTTGGCCGT | Reverse primer to amplify Toll-5 5'HR, with NotI cutting site |
| 17 | Toll-5 3' HR F AscI | Toll-5 | AGGCGCGCCTgcgaaatgttaacctatttg | Forward primer to amplify Toll-5 3'HR, with AscI cutting site |
| 18 | Toll-5 3' HR R | Toll-5 | TTGGGGGGTACCCCATAGATCCAGCAGCGTTAGA | Reverse primer to amplify Toll-5 3'HR, with SpeI cutting site |
| 19 | Toll-5 S BbsI gRNA | Toll-5 | gtcGTACAACGATGCGCCCGGTT | Toll-2 gRNA sense oligonucleotides for U6.3 |
| 20 | Toll-5ASgRNA | Toll-5 | aaacAACCGGGCGCATCGTTGTAC | Toll-2 gRNA anti-sense oligonucleotides for U6.3 |
| 21 | Toll1-Fwd | Toll-1 | CAACTGCCTACCAATCTCAC | with Toll1-Rev 2131-2422 of Toll CDS 292bp |
| 22 | Toll1-Rev | Toll-1 | CTATGAACACGCCCTTTTCC | With Toll1-Fwd 2131-2422 of Toll CDS 292bp |
| 23 | Toll2-Fwd | Toll-2 | GCAATATCGTCACAGCCTC | with Toll2-Rev 2718-3179 of 18W CDS 462 bp |
| 24 | Toll2-Rev | Toll-2 | CACACAAACTCGTAGTCCTTC | with Toll2-Fwd 2718-3179 of 18-W CDS 462 bp |
| 25 | Toll3-Fwd | Toll-3 | AATCACCTTCCAGCGAAAC | with Toll3-Rev 1084-1346 of Toll-3 CDS 263 bp |
| 26 | Toll3-Rev | Toll-3 | CCCAAAAACTTCAAAAACCCC | with Toll3-Fwd 1084-1346 of Toll-3 CDS, 263 bp |
| 27 | Toll4-Fwd | Toll-4 | CCTCATCTACTACACCTCCCTC | with Toll4-Rev 2766-3257 of Toll-4 CDS, 492 bp |
| 28 | Toll4-Rev | Toll-4 | TACGCCCTCAACTCGCTATC | with Toll4-Fwd 2766-3257 of Toll-4 CDS, 492 bp |
| 29 | Toll5-Fwd | Toll-5 | CTTAGCGACTTACTCAAGACC | with Toll5-Rev 313-670 of Toll-5 CDS, 358 bp |
| 30 | Toll5-Rev | Toll-5 | TCCCGAATGACACTATACCC | with Toll5-Fwd 313-670 of Toll-5 CDS, 358 bp |
| 31 | Toll6-Fwd | Toll-6 | CCTGAACGACAACCTGATAAC | with Toll6-Rev 2067-2449 of Toll-6 CDS, 383 bp |
| 32 | Toll6-Rev | Toll-6 | ACTCACAGCAATGGCAAAC | with Toll6-Fwd 2067-2449 of Toll-6 CDS, 383 bp |
| 33 | Toll7-Fwd | Toll-7 | CTCGCACAATCGCATCACAG | with Toll7-Rev 1914-2280 of Toll-7 CDS, 386 bp |
| 34 | Toll7-Rev | Toll-7 | GACGCAGACCACTCAAAGGA | with Toll7-Fwd 1914-2280 of Toll-7 CDS, 386 bp |
| 35 | Toll8-Fwd | Toll-8 | GCAGATCCTTAACCTGTCCC | with Toll8-Rev 1503-1946 of Toll-8 CDS, 444 bp |
| 36 | Toll8-Rev | Toll-8 | TTCCTCACCAAATCCACCC | with Toll8-Fwd 1503-1946 of Toll-8 CDS, 444 bp |
| 37 | Toll9-Fwd | Toll-9 | CCCCTACCTATCCTACAACATC | with Toll9-Rev 1239-1582 of Toll-9 CDS, 344 bp |
| 38 | Toll9-Rev | Toll-9 | AATCCAATCGCTCAAAGTCC | with Toll9-Fwd 1239-1582 of Toll-9 CDS, 344 bp |
